# Supplementary material for: Mid-infrared coincidence measurements on twin photons at room temperature
Source: Nat Commun. 2017 May 15;8:15184. doi: 10.1038/ncomms15184 (PMC5440726; doi:10.1038/ncomms15184)
Supplement: Supplementary Information — Supplementary Figures, Supplementary Notes and Supplementary References. [file ncomms15184-s1.pdf]

Supplementary note 1

In Supplementary Figure 1, the experimental characterization of the spectral response of the up-converter module for the 21.5 μm poling period is presented for two different crystal lengths.

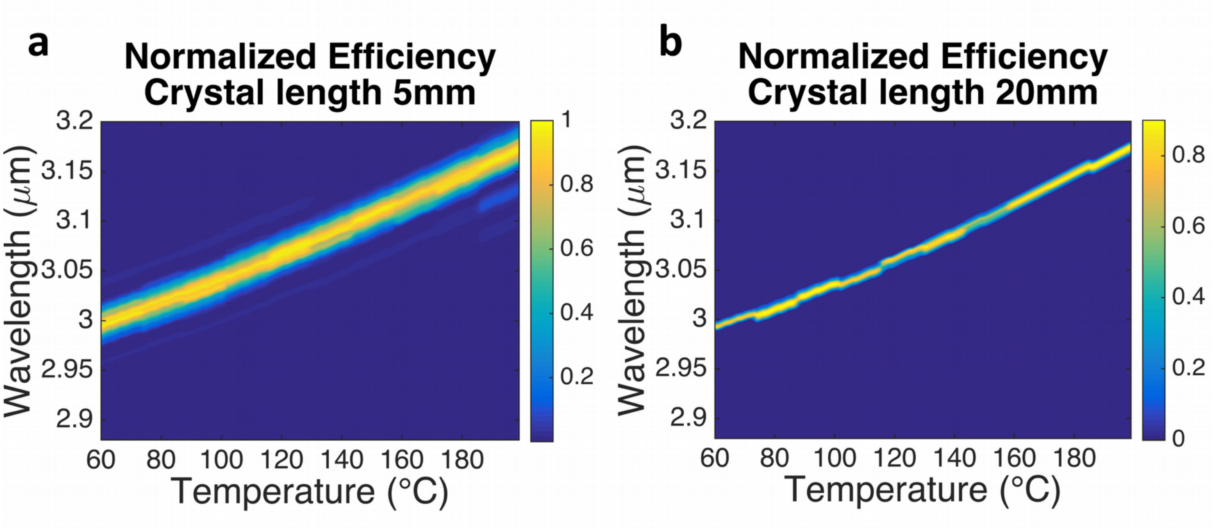

Supplementary Figure 1. **Spectral response of the up-converter module.**
(a,b), Comparison of the bandwidth for the 5 mm long and 20 mm long crystals for the 21.5 μm poling period.

In Fig. 3a of the main text, the phase matched wavelength and the relative bandwidth for different poling periods as a function of the crystal temperature are reported. The crystal length is 5 mm. As expected from the theory, longer phase matched wavelengths are associated to broader up-conversion bandwidths. Moreover, in Supplementary Figure 1(a,b), the differences in the bandwidths can be seen. The bandwidth of the 20 mm long crystal is narrower than the one of the 5 mm long, as expected. However, the two crystals, with the same nominal poling period 21.5 μm, present the same phase-matching relation as a function of the temperature. The very same considerations hold for all the other poling periods of the nonlinear crystals.

In Supplementary Figure 2, the output spectrum from the up-converter module is reported. This is the main source of noise for the SPAD and influences the SPAD dark counts. The measure was taken for the poling period equal to 21.5 μm (the same as Supplementary Figure 1(a,b), at a temperature equal to 100°C. In Supplementary Figure 2, the different curves refer to different values of the injected current intensity in the diode laser, which pumps the up-converter module.

The higher the injected current, the higher the intensity of the 1064 nm laser light that circulates within the module and, so the higher the conversion efficiency. But at the same time, the higher the current, the higher also the noise that comes from the up-converted black body radiation from the environment, as is highlighted by the black box in Supplementary Figure 2. A trade-off has been found in term of signal to noise ratio, at a current value of 3.6 A.

The measurements in Supplementary Figure 2 were performed using a filter in front of a visible monochromator to cut off the pump at 1064 nm. It is clearly possible to see both the contribution of the residual of the diode laser pump at 880 nm and of the result of the Second Harmonic Generation in the PPLN crystal of the 1064 nm pump, at 532 nm. In the region, which goes from 600 nm to 700 nm, the spectral lines that appear are due to stray-light from the room illumination. Other interesting part of the spectrum lies in the range which goes from 790 nm to 810 nm, which in fig. 2 is highlighted by a rectangle. The appearance of this spectral band is associated to the up-conversion of the black body radiation emitted by the environment. The band is wider than expected, and the reason lies in the fact that the black body radiation is isotropically emitted at every angle and the phase matching is angle dependent.

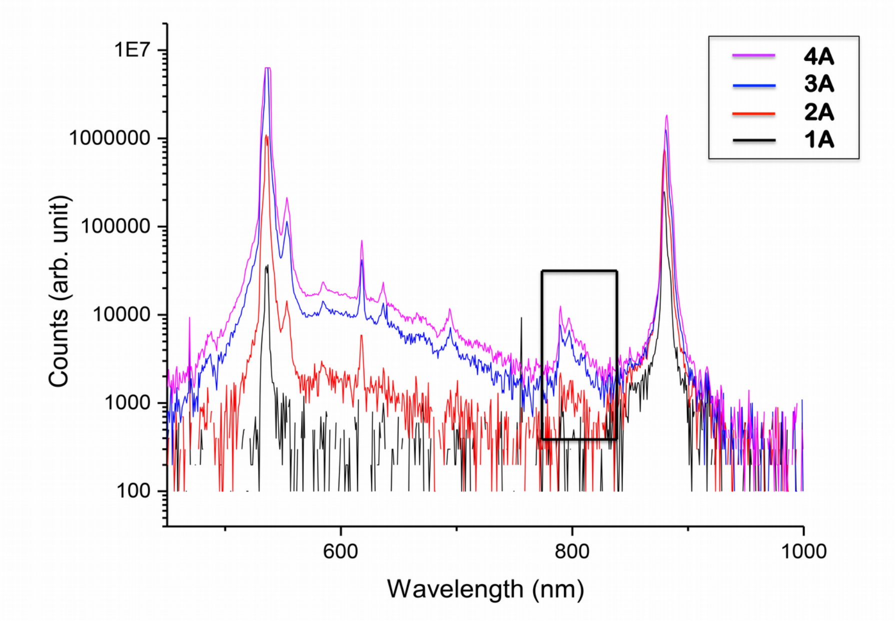

Supplementary Figure 2. **Spectra at the output of the up-converter module.**
The measure was taken for the 21.5 μm poled region, at a temperature of 100°C. The currents, which label the different curves, are the injected currents to the Broad Area diode Laser (BAL). The black box highlight the spectral region where the black body up-conversion may occur. The measure was taken with only a filter to cut off the pump at 1064 nm placed in front of a visible monochromator.

To reduce all these sources of dark counts when the up-converter module is coupled to the SPAD, a pinhole has been used to spatially filter out the noise. Moreover, since the up-converted photon pairs are actually generated in a very narrow band, bandpass filters have been used at the output of the up-converter module. In this way the noise is spectrally filtered, reducing the associated system background rate and increasing the signal to noise ratio. The filter transmittance for each set of filters is reported in Supplementary Figure 3. More details about the filters used after the module are discussed in the Methods section in the main text.

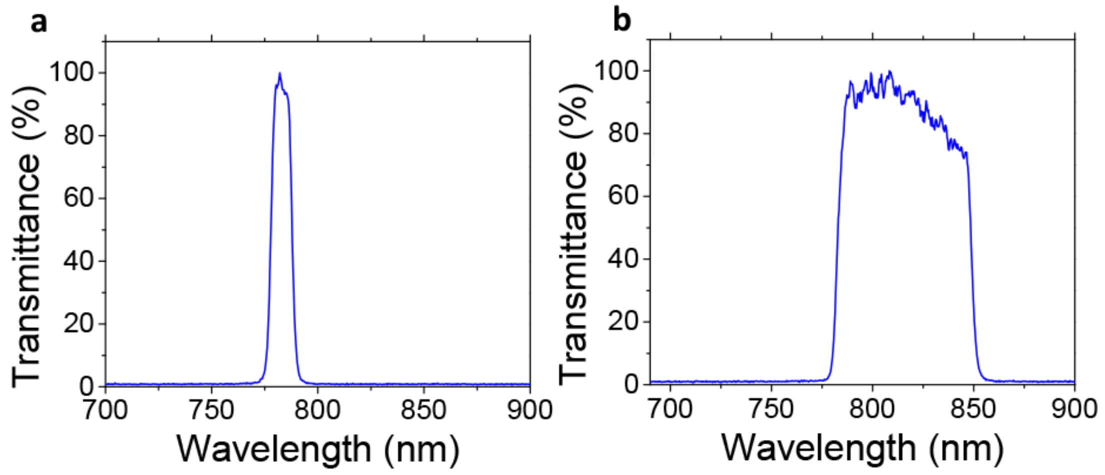

Supplementary Figure 3. **Filter transmittance for each set of filters placed after the up-converter module.**
(a) Transmittance of the set of filters used for the signal photons (centred at 778 nm). (b) Transmittance of the set of filters used for the idler photons (centred at 807 nm).

In Supplementary Figure 4 an experimental characterization of the CAR, which can be thought as the signal to noise ratio for a coincidence measurement, is reported. This measurement supports the choice of working with an injected current value of 3.6 A, as it gives the best CAR. Note that In Supplementary Figure 4, thermal effects within the module limits the conversion efficiency, which determine a CAR drop instead of a saturating behaviour for injected current values higher than 3.6 A.

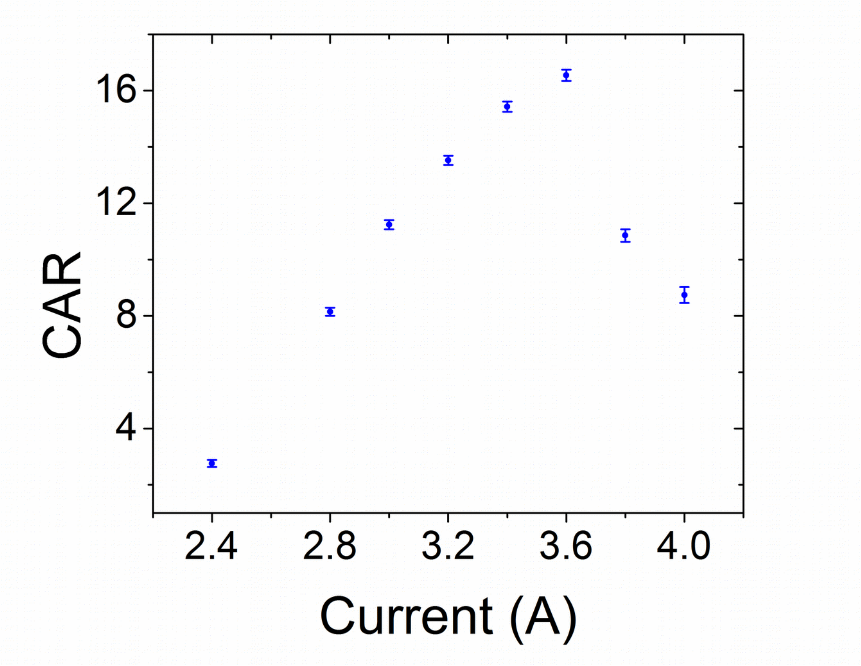

Supplementary Figure 4. **Coincidence to accidental ratio (CAR) characterization.**
CAR is reported as a function of the injected current to the BAL, within the up-converter module. The data were collected for 50 s of integration time. Error bars are derived from the standard deviation of the coincidence peak and the standard error of the mean accidental background rate.

Supplementary note 2

Starting from the experimental parameters, it is possible to estimate the coincidence peak due to the generated correlated pairs, versus the background of accidental coincidences [1,2].
In particular, knowing the incident photon flux on the detector and the losses from the generation stage, one is able to estimate how many correlated photon pairs arrive at the two detectors.
Indeed, the probability that both the photons of the pair are detected and not lost along the way, scales as the square of the losses. Instead, the probability that only one photon of the pair is detected, scales linearly with the losses. Actually, it is more likely to have mismatched couples at the detector, with respect to photon pairs. These single photons are not correlated in time and so, they may contribute to accidental coincidences at random delay between the two detectors, represented by the baseline in Fig. 4b. In addition, the accidental coincidences are also influenced by the dark counts of the detectors.
Therefore, if we call T the transmission amplitude from the generation to the detection stage, the experimental count rate subtracted for the dark count rate at each detector, C_{exp}, is given by

$$C_{\text{exp}} = NT \tag{1}$$

where N is the rate of generated pairs of photons. The correlated photons that arrive at each detector are NT^2 and the mismatched photons $(NT - NT^2)$. T is a coefficient that ranges from 0 to 1 (in our case T = 0.0032, which corresponds to -25 dB of losses).
Note that the description presented here is simplified for the sake of clarity. It does note take into account multi-pair emission and consider the coefficient T equal for the signal and the idler path.
The simulation of the random arrival on each detector is based on a Poissonian distribution. To generate random timings, it is enough to invert the cumulative distribution function for the well known Poissonian exponential distribution. If λ is the average flux of photons on the detector and we want to know the delay between one click and the next one at the detector, this is given by

$$t_{\text{next}} = \frac{-\ln(u)}{\lambda} \tag{2}$$

where u is a random number, uniformly distributed between 0 and 1.
Thus, given an average flux, we build random time arrivals within the observation window.
The simulated coincidence plot reported in Fig. 4b is obtained by performing the cross correlation between the time sequences of the two detectors, generated thanks to the relation in Supplementary Eq. 2. In the simulation the experimental electronic delay is not taken into account, therefore the simulated coincidence peak in Fig. 4b appears at zero time delay, instead of the 8 ns observed for the experimental measurement.

Supplementary references

[1] Takesue, H. & Shimizu K. Effects of multiple pairs on visibility measurements of entangled photons generated by spontaneous parametric processes. *Opt. Commun.* **283,** 276-287(2010).

[2] Azzini, S., *et al.* Ultra-low power generation of twin photons in a compact silicon ring resonator. *Opt. Express* **20,** 23100-23107 (2012).
